# Complications of unintentional dural puncture during labour epidural analgesia: a 10-year retrospective observational study

**DOI:** 10.1186/s44158-023-00127-1

**Published:** 2023-10-25

**Authors:** S. Poma, M. C. Bonomo, G. Gazzaniga, M. Pizzulli, A. De Silvestri, C. Baldi, F. Broglia, M. Ciceri, M. Fuardo, F. Morgante, S. Pellicori, E. M. Roldi, M. P. Delmonte, F. Mojoli, A. Locatelli

**Affiliations:** 1 Anaesthesia and Postoperative Intensive Care, Department of Anaesthesia and Intensive Care 3, I.R.C.C.S. Policlinic San Matteo Hospital Foundation, Pavia, 27100 Italy; 2 Department of Anaesthesia and Intensive Care, ASST Bergamo EST, Seriate Hospital, Seriate, Italy; 3 Department of Anaesthesia and Intensive Care 1, I.R.C.C.S. Policlinic San Matteo Hospital Foundation, Pavia, Italy; 4 Clinical Epidemiology and Biostatistics, Scientific Direction, I.R.C.C.S. Policlinic San Matteo Hospital Foundation, Pavia, Italy

**Keywords:** Labour analgesia, Postdural puncture headache, Unintentional dural puncture, Neuraxial analgesia, Labour complications

## Abstract

**Introduction:**

Unintentional dural puncture (UDP) occurs in 0.5–1.5% of labour epidural analgesia cases. To date, little is known about evidence of UDP-related complications. This work aimed to assess the incidence of intrapartum and postpartum complications in parturients who experienced UDP.

**Methods:**

This is a 10-year retrospective observational study on parturients admitted to our centre who presented UDP. Data collection gathered UDP-related complications during labour and postpartum. All women who displayed UDP received medical therapy and bed rest. An epidural blood patch (EBP) was not used in this population. Once asymptomatic, patients were discharged from the hospital.

**Results:**

Out of 7718 neuraxial analgesia cases, 97 cases of UDP occurred (1.25%). During labour, complications appeared in a small percentage of analgesia procedures performed, including total spinal anaesthesia (1.0%), extended motor block (3%), hypotension (4.1%), abnormal foetal heart rate (2%), inadequate analgesia (14.4%), and general anaesthesia following neuraxial anaesthesia failure (33.3% of emergency caesarean sections). During the postpartum period, 53.6% of parturients exhibited a postdural puncture headache, 13.4% showed neurological symptoms, and 14.4% required neurological consultation and neuroimaging. No patient developed subdural hematoma or cerebral venous sinus thrombosis; one woman presented posterior reversible encephalopathy syndrome associated with eclampsia. Overall, 82.5% of women experienced an extension of hospital stay.

**Conclusion:**

Major complications occurred in a small percentage of patients during labour. However, since they represent high-risk maternal and neonatal health events, a dedicated anaesthesiologist and a trained obstetric team are essential.

No major neurological complications were registered postpartum, and EBP was not performed. Nevertheless, all patients with UDP were carefully monitored and treated until complete recovery before discharge, leading to an extension of their hospitalization.

## Background

Unintentional dural puncture (UDP) is a known risk of neuraxial techniques, occurring in 0.5–1.5% of labour epidural analgesia [1–3]. According to some studies, approximately 50–60% of women experience postdural puncture headache (PDPH) after UDP [4, 5]. To date, adverse events represent potentially life-threatening conditions for parturients, and high-risk complications have been described [6]. Nevertheless, there is limited evidence of other UDP-related complications during labour, delivery, and hospital stay.

This study aims to determine the incidence of intrapartum and postpartum-related complications in parturients who experience UDP.

## Materials and methods

### Study population, duration, and setting

In this retrospective observational single-centre study conducted at the Obstetrics Department of I.R.C.C.S. Policlinic San Matteo Hospital Foundation in Pavia (Italy), we enrolled consecutive parturients who, between 1st January 2011 and 31st December 2021, experienced UDP after labour analgesia. The local ethics committee approved the study (Protocol 0103615/21), and it was reported according to the STROBE guidelines. Informed consent was obtained from every patient enrolled in the study.

### Objectives

The primary aim of this study was to investigate the incidence of UDP in our clinical practice and assess the prevalence of UDP-related complications during labour and hospital stay.

We considered UDP-related complications during labour: total spinal anaesthesia, extended or partial motor block (Bromage = 3–1/2), hypotension (systolic pressure 30% below baseline value), abnormal foetal heart rate secondary to maternal issues, inadequate epidural analgesia (visual analogue scale—VAS > 4 after 20 min from the drug administration), the occurrence of a second UDP while repositioning the epidural catheter, and the need to perform general anaesthesia for an emergency caesarean section due the failure of neuraxial anaesthesia.

We assessed UDP-related complications during the postpartum period as follows: subdural hematoma, cerebral sinus thrombosis, posterior reversible encephalopathy syndrome (PRES), PDPH (an orthostatic headache that deteriorates 15 min after standing and improves after 15 min in the supine position, occurring after a lumbar puncture) [7], atypical headache (nonposture related or persistent after 7 days from UDP), neurological symptoms (visual, auditory or vestibular disorders, vomiting), and need for neurological consultation and neuroimaging for atypical headache or the onset of neurological symptoms. Moreover, an extension of the hospital stay after 96 h due to persistent UDP-related symptoms and hospital readmission within 30 days after discharge were also considered UDP-related complications. As a hospital stay of approximately 96 h may be acceptable in our clinical practice, we opted to classify a prolonged stay as exceeding 96 h. According to the literature, adverse events are most likely to occur within 30 days following discharge, which is why we considered readmission during this period.

Finally, secondary research goals addressed in this study were the total length of hospital stay and the relationships between ADP, breastfeeding, and the need for psychological support during hospitalization.

### Unintentional dural puncture (UDP) diagnosis and treatment

Neuraxial analgesia was performed with the standard technique using an 18-G needle and liquid mandrel, sitting position, and level of insertion L2–L3/L3–L4. A test dose of lidocaine 2% 60 mg was administered through the epidural catheter to rule out subarachnoid intravascular placement.

Analgesia was maintained with a top-up epidural bolus at the patient’s request (VAS score > 4) with levobupivacaine 0.1% 20 mg plus sufentanil 10 µg until reaching 6-cm cervical dilatation and then with levobupivacaine 0.125% 25 mg.

The midwife on duty monitored the vital parameters of the parturient. Continuous cardiotocography (CTG) was used during labour for foetal monitoring. The anaesthesiologist assessed the resolution of pain and investigated the potential onset of any complications related to the procedure.

Dural puncture was diagnosed by cerebrospinal fluid (CSF) leakage through the dural hole created by the Tuohy needle or catheter placed for analgesia. In case of doubt, the liquid was examined through a strip test for glucose and proteins, and the positive outcome confirmed the presence of CSF.

Once the diagnosis of UDP had been confirmed, the anaesthesiologist shared it with the patient and the obstetric team and reported the complication on paper and in computerized files. Then, the team considered the following options, according to the patient’s request: the nonexecution of new analgesia, epidural catheter repositioning usually in the upper intervertebral space, or catheter placement in the subarachnoid space, especially in cases of a difficult procedure.

When the peridural catheter was repositioned, the antalgic dose was administered slowly in fractionated doses to achieve pain relief.

In the case of a subarachnoid catheter, analgesia was usually performed with levobupivacaine 0.1% 2.5 mg plus sufentanil 3 µg until reaching 6-cm cervical dilatation and then with levobupivacaine 0.1% 2.5 mg. However, in our reality, there was no established and agreed protocol for spinal catheter management during labour or anaesthesia in the case of caesarean section throughout the study.

The catheter was removed after 2 h of observation following the child’s delivery to prevent the risk of infection and allow the mother’s recovery in an unmonitored ward. No epidural or intrathecal bolus or infusion of saline solution was administered. All patients with UDP and postpartum PDPH were treated with oral or intravenous fluids to maintain the correct hydric balance, preventive anticoagulant therapy, and analgesics if VAS > 4 (paracetamol 3 g EV/day or nonsteroidal anti-inflammatory drugs). The presence of headache and symptoms while sitting or standing was assessed daily by the obstetric staff and anaesthetists. In case of headache during mobilization, bed rest was recommended for the next 24 h until gradual recovery in an upright position was achieved. Furthermore, a routine epidural blood patch (EBP) was not performed.

In cases of neurological symptoms and atypical headache, a complete examination was conducted by a neurologist, and further neuroimaging investigations (computed tomography scan, rachis, and brain magnetic resonance imaging) were arranged when deemed appropriate.

With dedicated ward staff, bonding between each new mother and her newborn was encouraged and supported, promoting breastfeeding and skin-to-skin contact. Moreover, psychological support was offered to mothers upon their request.

Patients were discharged after the complete resolution of symptoms (numeric rating scale score for headache < 3) and when the mother was able to manage her daily activity and her child’s daycare.

Women were advised to return to our hospital if a headache reoccurred.

### Data collection

Demographic and clinical data were stored through electronic medical records. The following characteristics were collected: maternal age, body mass index (BMI), parity, type of delivery (vaginal birth, instrumental, or C-section), preanaesthesia assessment, and analgesia technique (epidural analgesia or a combined technique). We gathered all data in an Excel file (Excel2010, v14.0, Microsoft Corporation, Redmond, WA, USA) that contained anonymized patient details.

### Statistical analysis

The incidence of UPD and the prevalence of UPD-related complications during labour and postpartum were calculated by an exact binomial confidence interval of 95% (95% CI).

## Results

During this study, 21,270 child deliveries occurred, and 7718 procedures for labour analgesia were performed (36% of deliveries). As a result, a total of 97 UDPs were recorded (1.25%, 95% *CI*, 1.01–1.53), 81 during labour (1.05%), and 16 postpartum (0.2%), with the typical onset of PDPH. In those UDP cases detected during labour, 47 patients underwent epidural catheter repositioning, and 26 received subarachnoid analgesia. Only eight women decided to refuse any other type of analgesia. The clinical anamnestic characteristics of the population under examination are summarized in Table 1.

**Table 1:** Characteristics of women presenting UDP (*N* = 97)

	Average (SD)	*N* (%)
**Anthropometric characteristics**		
Age (years)	30.7 (5.4)	
BMI (kg/m^2^)	27 (3)	
**Obstetric characteristics**		
Parity		
Primiparous woman		70 (72.2)
Multiparous woman		27 (27.8)
Childbirth delivery		
Vaginal delivery		69 (71.1)
Assisted vaginal delivery		13 (13.4)
Caesarean section		15 (15.5)
**Anaesthesia characteristics**		
Technique		
Epidural analgesia		89 (92)
Combined technique		8 (8)
Assessment prior to the procedure		93 (95.8)
Post UDP strategy		
Nonexecution of analgesia		8 (8.2)
Repositioning of the epidural catheter		47 (48.5)
Subarachnoid analgesia		26 (26.8)
Unknown UDP		16 (16.5)

### Prevalence of UDP-related complications during labour and hospital stay

After the diagnosis of UDP, 28 patients (28.8%) did not experience further complications either in labour or postpartum. On the other hand, 69 women (71.2%, *CI* 95%, 0.61–0.79) presented UDP-related complications as follows: 15.5% (15/97) during labour, 39.2% (38/97) during postpartum, and 16.5% (16/97) during both.

### UDP-related complications during labour

During labour, 31% (31/97, *CI* 95% 0.22–0.422) of patients experienced complications (Table 2).

**Table 2:** UDP complications during labour (*N* = 97)

**UDP-related complications during labour**	*N* (%)
Total spinal anaesthesia	1 (1)
Extended motor block (Bromage = 3)	3 (3)
Partial motor block (Bromage = 1/2)	5 (5.1)
Hypotension	4 (4.1)
Inadequate or failed analgesia	14 (14.4)
Foetal heart rate abnormalities secondary to maternal issues	2 (2)
Second unintentional dural puncture	2 (2)
Conversion to general anaesthesia for caesarean section	5/15^a^ (33)

^a^15 = number of caesarean sections

In only one case, undiagnosed UDP progressed in total spinal anaesthesia during the conversion to epidural anaesthesia for C-section, with hypotension and desaturation. The adverse event was overcome without any maternal or neonatal harm.

Three percent of patients (3/97) showed extension of locoregional anaesthesia with a motor block (Bromage = 3) after a test dose of lidocaine, and in one case, instrumental delivery was performed.

A total of 5.1% of patients presented partial motor block (Bromage = 1/2) after administering the epidural antalgic dose according to the protocol. The epidural catheter was repositioned in all these patients following dural puncture.

Fourteen patients with UDP presented inadequate or failed analgesia: 30% with spinal catheters and 12% with epidural catheter repositioning.

Two women showed an abnormal foetal heart rate (FHR) pattern secondary to maternal issues, and in one case, upon the occurrence of bradycardia, a C-section was performed.

Among 15 patients who underwent an emergency C-section, 5 (33%) needed conversion into general anaesthesia after an unsuccessful attempt at spinal anaesthesia (4 parturients) or the failure of epidural catheter repositioning (1 parturient).

### UDP-related complications during postpartum

In this study, 55% of patients (54/97, *CI* 95%, 0.45–0.55) experienced postpartum complications. However, none of the women developed a subdural hematoma or a cerebral venous sinus thrombosis.

One preeclamptic patient presented PRES associated with eclampsia 60 h after UDP, although without PDPH.

One patient suffered from emesis and neck pain without PDPH.

A total of 53.6% of the patients experiencing UDP (52/97) presented postpartum headaches: 77% of them (40/52) suffered from a typical headache, while 23% (12/52) experienced atypical cephalalgia.

Neurological symptoms were reported in 13.4% of women (13/97). A neurological consultation for atypical headaches or the onset of symptoms was required in 14.4% of cases (14/97). A brain/spine CT scan or MRI was performed in all patients after neurological evaluation; in 5 women, the test was negative, while 8 presented with the typical features of intracranial hypotension. Moreover, in parturients who experienced eclampsia, neuroimaging showed findings compatible with initial signs of PRES.

In this study, 82.5% of women (80/97) had an extended hospital stay (> 96 h) due to the persistence of UDP-related symptoms. However, after discharge, no patient was readmitted over the following 30 days.

The principal postpartum complications are summarized in Table 3.

**Table 3:** UDP complications during postpartum (*N* = 97)

**UDP-related complications during postpartum**	*N* (%)
Subdural hematoma	0
Cerebral venous sinus thrombosis	0
PRES	1 (0.9)^a^
PDPH	52 (53.6%)
Neurological symptoms	13 (13.4)
Neurological consultant	14 (14.4)
Intracranial hypotension on neuroimaging	8 (8.2)
Hospital stay > 96 h	80 (82.5)

^a^Preeclamptic patient

### Secondary goals

Patients who presented UDP had an average hospital stay of 6 days (SD 2.3). Furthermore, 8 women (8.2%) with PDPH chose not to breastfeed. Finally, only 2 women (1.9%) requested psychological support during their hospital stay.

## Discussion

In this study, conducted at I.R.C.C.S. Policlinic San Matteo Hospital Foundation in Pavia (Italy), the incidence of UDP appeared to be 1.25%, and the result is in line with the literature. Moreover, since our centre is part of the University of Pavia, trainees’ presence reasonably influences UDP incidence (0.6% specialists vs 3.1% trainees) [8].

According to our results, 31 patients (31%, *CI* 95% 0.22–0.422) developed adverse events during labour.

Among the complications, the incidence of total spinal anaesthesia was 1 over 7787 analgesia procedures (0.01%), which is lower than what is reported in the literature (0.02%) [9]. In our analysis, this event occurred during the conversion to anaesthesia for an emergency C-section. Since this procedure is often conducted in a challenging setting, the risk of undiagnosed UDP and total spinal anaesthesia is very likely due to a higher dose of local anaesthetic.

Three out of 97 patients (3%) with undiagnosed UDP experienced extensive motor block after performing a test dose of lidocaine. Therefore, it would be reasonable to assume that titration of the analgesic dose might be associated with fewer complications [10]. Moreover, after UDP and repositioning of the epidural catheter, 5.1% of parturients presented a partial motor block. So, in light of the possibility of CSF leakage through the gap of the dura mater, drug administration in smaller doses might also be recommended in these scenarios, and a reduction based on the patient’s clinical response may be a sensible option [11].

A total of 14.4% of parturients with UDP presented inadequate analgesia, and these data are consistent with previous studies [12]. Nevertheless, the rate rises to 30% after considering subarachnoid catheter cases. This seems to contradict the most recent findings, which reported no differences in the quality of analgesia with a spinal catheter or after repositioning a peridural catheter [13]. However, the lack of a structured protocol for analgesia with spinal catheters and the fear of drug-related complications in our clinical practice may have influenced this aspect.

In the literature, 33% of women undergoing an emergent C-section required general anaesthesia, although this percentage is higher than our reality (2% of general anaesthesia for C-sections), where this event is primarily due to subarachnoid anaesthesia failure. Other studies show a significant risk of a failed spinal catheter during the conversion of analgesia to C-section anaesthesia [14]. The reasons are unclear, although an anaesthetic leakage through the breach or an underdosing to avoid side effects (i.e., extensive motor-sensory blockade) may have a role in the quality of anaesthesia.

Over the postpartum period, 53.6% of patients developed headaches. Headaches are a common postpartum symptom and can be related to several other causes; the most common are tension headaches, followed by preeclampsia headaches and neuraxial anaesthesia-related headaches [15]. Therefore, during pregnancy, a preanaesthesia evaluation conducted by an anaesthesiologist plays a pivotal role in women’s education, leading to the earlier detection of symptoms.

In this study, 95% of women underwent a prepartum assessment and were informed about analgesia complications.

As argued by several case reports in the literature, major UDP-related events could lead to fatal outcomes if not readily diagnosed. In a large retrospective cohort study, the incidence of subdural hematoma and cerebral venous thrombosis was higher in women with PDPH, and these complications were usually identified after discharge from the hospital with a median time to readmission of 5 days [16]. The association of UDP-related headaches with a subdural hematoma can cause significant morbidity and mortality (7%). Furthermore, UDP has been reported as a risk factor for cerebral venous thrombosis. During puerperium, the chance of developing this complication increases due to the fluctuation of intracranial pressure during delivery and the hypercoagulability state of pregnancy. In addition, in the case of UDP, the leakage of CSF might lead to hypotension and subsequent vasodilation, thus promoting the development of thrombosis [17].

In our experience, no patient developed subdural hematoma or sinus thrombosis. Nevertheless, clinicians should be aware of the possibility of these complications and pay attention to the onset of atypical signs and symptoms of PDPH [18, 19].

In this study, a pre-eclamptic patient showed PRES on MRI and presented generalized tonic‒clonic seizures without PDPH development. Several cases of UDP-related PRES have been reported in the literature, although it is still unclear whether the delayed treatment of PDPH may trigger PRES or whether the diagnosis of UDP may lead to a misdiagnosis of PRES by neglecting differential diagnoses [20, 21].

As previously mentioned, in this study, women were treated with conservative medical therapy and bed rest, and EBPs were not performed.

This aspect represents a peculiarity in our population of patients. During our study (2011–2021), a clear indication for using EBP supported by randomized controlled trials was absent, especially regarding its role in PDPH prevention and efficacy and its timing, applications, and management [22].

According to the Obstetric Anaesthetists’ Association’s (OAA) new guidelines of 2019 [23, 24], EBP was considered the gold standard in UDP patients, and it was recommended in cases of particularly persistent and debilitating headaches nonresponsive to conservative therapies [24]. However, in Italian clinical practice, EBP is only used in 25% of cases, a smaller percentage than that in European practice (60%) [25]. The risk of side effects, the chance of failure (30%) or partial success (38%) [26], and the lack of evidence supporting EBP as a protective strategy against the development of major neurological complications [27] may have influenced its use in our clinical practice. Many other treatments have been tested over the years, including pharmacological options such as caffeine, theophyllines, adrenocorticotropic hormone, steroids, and triptans, as well as invasive procedures like acupuncture, greater occipital nerve or sphenopalatine ganglion blocks, epidural morphine, and epidural or spinal fluid administration. However, more evidence still needs to be produced to make firm recommendations, and further investigations are required to understand their effectiveness.

Conservative treatment with analgesia, hydration, and bed rest in a supine position with preventive anticoagulant therapy may provide pain relief and control the symptoms. Increasing fluid intake may enhance the production of cerebrospinal fluid, and immobilization may reduce hydrostatic pressure in the subarachnoid space and help seal the dura, reducing CSF leakage. Nevertheless, randomized controlled studies are lacking to prove the efficacy of these strategies in the resolution of PDPH, especially those concerning prolonged bed rest, which is not recommended in current guidelines [28]. However, no major complications were identified in our study, despite not using EBP and limiting the therapy to a medical approach. Nevertheless, 96% of women suffering from UDP had an extended hospital stay due to longer assistance and monitoring to promote their recovery and to check for the occurrence of other complications that usually arise a short time after early discharge. In this regard, home monitoring is only occasionally feasible and suitable.

### Study limitations

The retrospective analysis, the monocentric nature of the investigation, and the limited sample size represent limitations to this study and are also related to the low incidence of the phenomenon. Moreover, other UDP effects concerning the delivery type or the labour duration have not been evaluated, although a prolonged stage II has already been suggested [29]. Finally, although the onset of long-term complications such as chronic headaches, postpartum depression, and posttraumatic disorders has not been investigated, it may be a potential subject of further research [30].

## Conclusions

Major UDP-related complications, such as total spinal anaesthesia and extensive motor block, occurred in a very low percentage of analgesia performed during labour. Nevertheless, a dedicated anaesthesiologist and a trained obstetric team during these circumstances are crucial, given the high risk of potentially dangerous events for the mother and the newborn.

Our study revealed a significant rate of failure in subarachnoid anaesthesia and analgesia following UDP. It highlighted the necessity for shared clinical protocols in the management of spinal catheters.

Although EBP was not used, no major neurological complications were recorded during the postpartum period. It should be noted that in our study, all patients suffering from UDP were carefully monitored and treated with medical therapy until complete recovery, even if that meant a more extended hospital stay.

## Data Availability

The datasets used and/or analysed during the current study are available from the corresponding author upon reasonable request.
